# ASPIRE-ing to Excellence at SIUSOM

**DOI:** 10.15694/mep.2017.000082

**Published:** 2017-05-05

**Authors:** Anna T. Cianciolo, Debra L. Klamen, Austin M. Beason, Evyn L. Neumeister

**Affiliations:** 1 Southern Illinois University School of Medicine

**Keywords:** Educational innovation, Quality improvement, ASPIRE Awards, Undergraduate medical education

## Abstract

This article was migrated. The article was marked as recommended.

This Personal View article describes the experience of Southern Illinois University School of Medicine (SIUSOM) with the AMEE School Programme for International Recognition of Excellence in Education (ASPIRE) awards program. Institutional leaders considering applying may need something more than the program description to take the plunge. We use narrative to present our reasons for applying, how the application and review process went for us, and the benefits of getting involved. By sharing our story, we hope to motivate other educators who believe in their school’s educational excellence to visualize themselves as applicants and take action.

## Introduction

It was 2012 when Debra Klamen, MD, MHPE, first learned of the AMEE School Programme for International Recognition of Excellence in Education (ASPIRE) awards. As Associate Dean of Education and Curriculum and Chair of Medical Education at Southern Illinois University School of Medicine (SIUSOM), Deb was in the midst of carrying out her charge to promote curriculum innovation and reestablish SIUSOM’s reputation as a leader in education. She encountered the ASPIRE program details online while exploring ways to get involved in the international medical education community. At the time, ASPIRE was a brand new program, introduced to recognize medical, dental, and veterinary schools for educational excellence in ways that conventional school rankings and accreditation standards did not [ Harden & Wilkinson, 2011]. There were three areas of recognition: Student Assessment, Student Engagement, and Social Accountability. The program’s intent and criteria, its international scope, and its three areas of focus all captured Deb’s interest. Deb submitted the application on behalf of SIUSOM, and in August 2013, our school was recognized at the AMEE conference in Prague for excellence in all three areas.

There is, of course, a bit more to the story than that. For one, there are several institutions who also have received ASPIRE awards ( http://www.aspire-to-excellence.org/ASPIRE+Award+Winners/), including Leeds (United Kingdom) and Aga Khan (Pakistan), who have won multiple awards, and the Leaders in Indigenous Medical Education (LIME) Network (Australia), who have published a detailed account of their program [see Mazel & Ewen, 2015], among others. In this narrative article, we describe why we applied, how the application and review process went for us, and the benefits we continue to enjoy because we got involved. We believe that details of schools’ experience with the program may be of interest to institutional leaders considering applying, but who need something more than the online program description ( https://amee.org/awards-prizes/aspire-award) to take the plunge. We hope that our illustrative example will motivate other educators who believe in their school’s educational excellence to visualize themselves as applicants and take action. Available in MedEdPublish, we also hope this article will prompt other ASPIRE winners to share their stories of extraordinary engagement in teaching and learning. Through our sustained involvement with the ASPIRE program, we have come to believe that orienting community attention on matters of educational excellence will promote it worldwide; this conversation is one important step in that direction.

## Why we applied

Founded in 1970, SIUSOM is a small, community-based medical school located in the Midwestern United States. Our institutional mission is to assist the people of central and southern Illinois in meeting their health care needs through education, patient care, research, and service to the community. Recognizing education as our top priority, SIUSOM has been on the leading edge of educational innovation since its establishment. For example, in 1972, SIUSOM launched MEDPREP, one of the first post-baccalaureate pipeline programs to promote access to professional education for disadvantaged and minority students [ Jackson, McGlinn, Rainey, & Bardo, 2003]. In 1976, we were the first medical school to establish a complete set of goals and objectives for the medical degree [ Silber, Williams, Paiva, Taylor, & Robinson, 1978]. With the recruitment of Howard Barrows, MD, from McMaster University in 1981, we began a pioneering journey of innovation in pre-clinical instruction [Barrows & Tamblyn, 1980; Distlehorst, Dawson, Robbs, & Barrows, 2005; Distlehorst & Robbs, 1998] and clinical performance examination, to include standardized patient exams [ Williams, Barrows, & Moy, 1987; Williams, Barrows, Vu, Verhulst, Colliver, Marcy, & Steward, 1987], progress testing of clinical reasoning [ Williams, Klamen, & Hoffman, 2008; Williams, Klamen, White, et al., 2011], and written assessment of diagnostic justification [ Cianciolo, Williams, Klamen, & Roberts, 2013; Williams & Klamen, 2012]. Most recently, with support from the Josiah Macy, Jr. Foundation, we have reformed our clerkship curriculum to address the limitations of today’s clinical learning environment for developing trainees’ clinical skills [ Klamen, 2015].

We share this brief history to illustrate how institutions like SIUSOM represent the type of medical school that is above-standard on accreditation criteria (SIUSOM has been citation-free for two consecutive Liaison Committee on Medical Education accreditation reviews) yet below the radar on national rankings. Although our faculty maintain active and ground-breaking research programs and clinical practices, our institutional investment and risk-taking, time and again, has emphasized educational innovation. In addition, consistent with our founding charge [see Jackson et al., 2003], our student body represents the community we seek to serve; roughly half of our students come from small, rural hometowns (population 25,000 or less), and approximately 10% are racial/ethnic minorities underrepresented in medicine. Our small size, rural location, community emphasis, and prioritization of educational equity lower our standing on the criteria against which medical schools typically are ranked, including research funding and entrance exam scores. The ASPIRE selection criteria were perfectly fit to our school because they were designed to recognize outstanding performance in education while accounting for the reality that schools vary a great deal in their local circumstances and adapt their approach to education accordingly [ Harden & Wilkinson, 2011].

For these reasons, Deb was confident SIUSOM should apply for an ASPIRE award. Well, not just one award. In its pilot year of 2012, the program required no fee to submit an application; true to her go-getter spirit and empowered by no-cost submission, Deb submitted an application for SIUSOM in all three areas. Importantly, Deb was also motivated by the belief that participating in the global medical education community had value. She knew that SIUSOM could contribute to international dialogue on improving education, but she also recognized that we would benefit tremendously from re-examining our own ideas from perspectives formed outside of U.S. national policies and concerns. Many celebrated leaders in medical education do not reside in the U.S., and Deb saw that it was time to make a serious effort to stand and learn among them.

## The Application and Review Process

For us, preparing ASPIRE award applications was a fairly straightforward process of documenting in detail what we do. There was a lot to say (our application for Student Assessment was 93 pages long!), but our thoughts were organized already by our mission-centered, continuous quality improvement approach to medical education. That is, our applications reflected the conversations about excellence we were already having among faculty, staff, and students via curriculum committee meetings, evaluation surveys and focus groups, accreditation self-analysis, journal clubs, and our annual, schoolwide Symposium for Teaching and Learning. Importantly, our applications also reflected the scholarly dialogue we were having with the broader community through conference presentations, journal articles, and innovation grant proposals. Applying for ASPIRE awards allowed us to share our ongoing discussions with people dedicated to reviewing our progress according to thoughtfully prepared standards for educational excellence.

We have now had experience applying for ASPIRE awards successfully and unsuccessfully, as part of the pilot program and as part of the program that has emerged since then. (When Faculty Development was added to the areas for recognition in 2015, we submitted a fourth application. We were not selected for an award, but were invited to resubmit our application.) Each time, we have found the submission process to have all the features one would hope it to have. Specifically, the ASPIRE website ( http://www.aspire-to-excellence.org) clearly lays out the eligibility criteria and application process and presents the straightforward, evidence-based standards that the evaluation panel uses to judge each area of excellence. There now is a £2,500 application fee per area of excellence, but it is reduced for institutions applying for an award in more than one area and institutions situated in emerging economies.

We have also found the review process to be constructive and fair. Regardless of whether we received an award, the feedback on our application was of high quality and provided helpful guidance on the strengths and weaknesses of our performance in the application’s area of excellence. In addition, the recently established ASPIRE Academy, comprising representatives from ASPIRE award-winning institutions, provides a variety of resources to enhance the likelihood of success on the first try. These resources include workshops delivered at the AMEE conferences ( https://amee.org/conferences) and MedEdWorld webinars ( https://www.mededworld.org/Webinars/Upcoming-Webinars.aspx). In sum, the process of applying for an ASPIRE award is not complicated or difficult; the greater challenge is engaging in the long-term institutional self-examination and improvement process that promotes and sustains educational excellence. Once that challenge is mastered, the application is easy and the feedback is invaluable to quality improvement.

## Benefits of Submission

Reflecting on our experience, we believe that submitting an ASPIRE award application has several benefits. First, it provides unparalleled opportunity to focus your institution’s self-development on excellence. That is, institutional leaders seeking excellence have the ASPIRE criteria to use as objectives, and the award program itself helps them justify the application of resources to improvement planning, monitoring activity, progress evaluation, and adjusting the approach as needed. Of course, accreditation standards should also stimulate continuous quality improvement like this. However, schools that meet accreditation standards may vary a great deal in their emphasis on educational growth and innovation; schools that push the envelope on excellence set the pace for accreditation standards, yet they may be indistinguishable from other schools when measured against them [ Harden & Wilkinson, 2011]. Submitting an ASPIRE award application stimulates an institutional growth mindset of continuous learning [ Dweck, 2008] and helps leaders commit resources to going above and beyond accreditation standards. This mindset already existed at SIUSOM (Deb is often heard at curriculum committee meetings saying: “If it ain’t broke, make it better!”), so applying for ASPIRE awards validated our priorities and values. Other schools with this mindset also will appreciate the opportunity to share their story, but for some schools, the ASPIRE program may be just what is needed to get the ball rolling past accreditation and on to excellence.

A second benefit of submission is educational community development at the institutional level and beyond. Preparing an application requires your school to reflect on what it is doing relative to the excellence criteria. Regardless of the area of excellence, the criteria are comprehensive and systemic in nature, so evaluating and documenting your school’s excellence must involve input from a diverse array of stakeholders, including faculty, students, and, in some cases, patients and community members. This reflection promotes an optimistic sense of common cause among stakeholders and an orientation toward the future for your institution. Consideration of the ASPIRE excellence criteria also requires engagement with the scholarly community, as institutional structures and practices that meet evidence-based standards must themselves be evidence-based. Moreover, some of the ASPIRE excellence criteria call for scholarly productivity as demonstration of broader impact. When the preparatory work is done, submitting an application brings your school to the attention of international observers, whose constructive feedback gives you a sense of your institution’s place in the international community and ideas for where it can go further. For us, the act of submitting started forward-looking conversations with new friends and energized our involvement in the global community of health professional educators.

Finally, the program’s application fee gives your institution permission to set time aside and focus on itself. The mere existence of a fee gives the hopeful applicant pause to think carefully about whether the time is right to submit. Initial review of the excellence criteria, ASPIRE Academy resources, and readily available information about your own institution should help you judge whether submission is a wise investment or whether you should wait until you can prepare a stronger application. When you do choose to apply, the fee once again gives you a reason to take seriously the application process; now that resources have been committed, building the argument for your institution’s excellence carries new weight. The application fee may help you protect time to meet with key stakeholders and keep your office door closed while you write. The application fee has influenced us in these ways as we consider resubmitting our application for the Faculty Development award and applying for an award in the newest area of excellence: Simulation.

## Benefits of Recognition

The benefits of being recognized for excellence seem obvious. It is gratifying to feel rewarded for having made learners and learning-your passions-a top priority. It means something to know that a global organization shares your values and thinks your efforts reflect excellence. It is exciting to take the stage in front of thousands of AMEE conference attendees, aware that your school’s educational quality is the center of everyone’s attention, at least for a few moments. The ASPIRE award trophies are artfully designed, and they make an impressive display in public spaces. It is energizing to realize that, in being recognized, you now have greater capacity to assist in other institutions’ journey toward excellence and, in so doing, to advance the field of health professional education as a whole.

**Figure:**
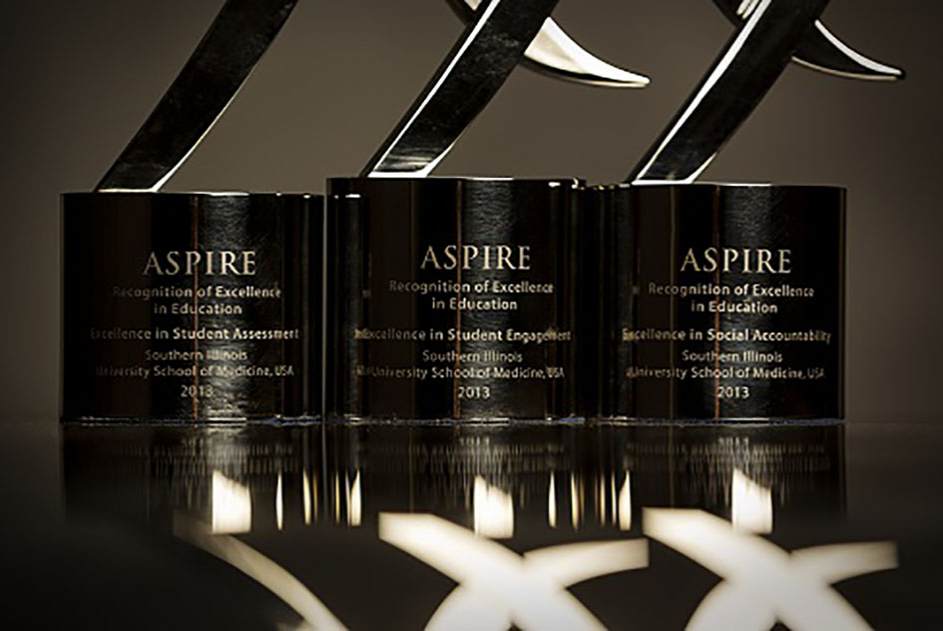
SIUSOM 2013 ASPIRE Awards: Student Assessment, Student Engagement, & Social Accountability (Photo credit: Jason Johnson, Southern Illinois University School of Medicine)

But, here too, there is more to the story. Within our institution, receiving ASPIRE awards has mobilized broader effort to sustain excellence, and now a more diverse range of people are looking around to see how they personally can “make it better.” Basic science and clinical faculty are collaborating more closely with medical education faculty to apply learning principles to curriculum design, from flipped classroom delivery to a multi-disciplinary PhD program in population health sciences. Beyond offering their perspectives and opinions, our students have become an integral part our educational mission, co-leading curriculum design and development and the expansion of social outreach to our local community. Both students and faculty have become decisively more engaged in educational research, adopting new methods of inquiry, disseminating their findings, and finding their place among national and international scholars. On a basic level, educational excellence has become the center of more conversations that are more inclusive and have greater impact both within and outside of our institution.

**Figure:**
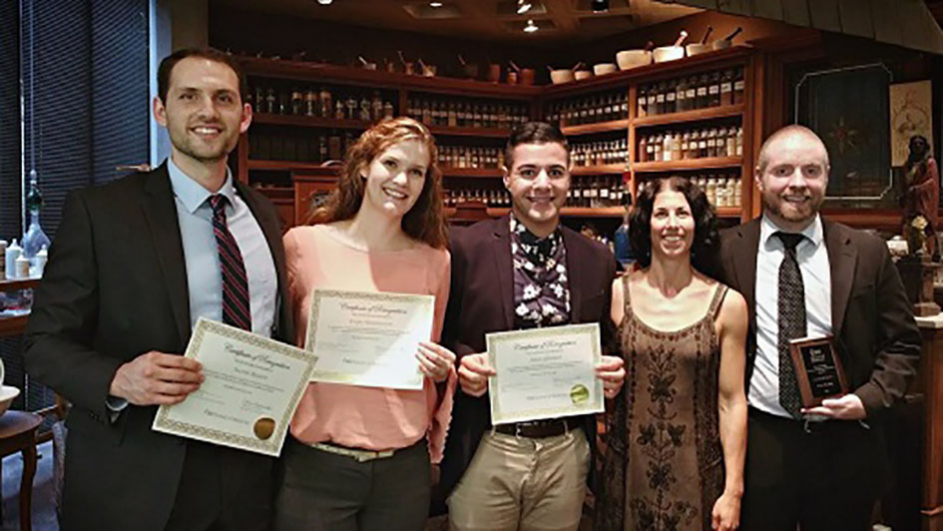
SIUSOM 2016 Leading Student Educators (left to right), Austin M Beason, Evyn L Neumeister, Allen Ghareeb, (Anna T Cianciolo, Medical Education faculty), and Sean Murray (Photo Credit: Anna T Cianciolo)

The ASPIRE awards, proudly presented in a glass case just below our school’s mission statement in the Dean’s lobby, guide this effort by communicating who we are. The centrality of educational excellence to our mission and the value we place on community participation (local, national, and international) are clearly visible to all faculty, staff, students, visitors, and applicants who pass through. Having won the awards protects our institutional culture of learning by conveying our values to others, helping us recruit likeminded faculty and students, and demonstrating that efforts to innovate matter outside the institutional boundaries. All of this is especially meaningful to a medical school located in a place like Illinois, where financial woes threaten higher education funding; by energizing the spirit of innovation and mobilizing institutional resources in a way that facilitates accreditation, stimulates growth, shapes culture, and builds national and international reputation, ASPIRE awards expand the value-added argument for investing in education.

**Figure:**
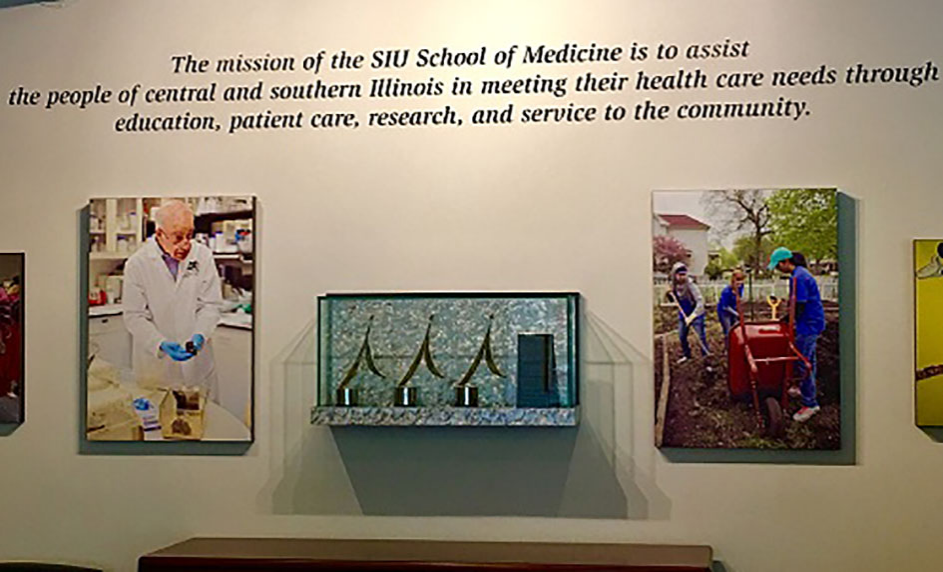
2013 ASPIRE Awards in the SIUSOM Dean’s Lobby (Photo Credit: Anna T Cianciolo)

## Closing Reflection

We hope that readers who made it to the end of this essay better understand what the ASPIRE program is all about and the impact it had on one participating school. Although each institution’s story is unique, the accumulation of narrative accounts may increase the likelihood of common benefits. We would be delighted to see other ASPIRE award recipients share their story and advice, as we are hopeful that our collective experience will help award applicants to better chart their own path to excellence and recognition. But perhaps the key test is whether we would take our own example: Would we do it again? Yes! We plan on it!

## Take Home Messages

• The ASPIRE Awards program offers schools that prioritize educational innovation an unparelled opportunity to be recognized for their values and effort.
• The application process is not difficult, but success requires that institutions maintain a self-development process focused on excellence.
• Participating in the ASPIRE Awards program, regardless of outcome, fosters local, national, and international educational community development.
• The benefits of receiving an award go beyond being recognized for excellence; winning an award promotes striving for future excellence and expands the value-added argument for investing in educational innovation.

## Notes On Contributors

**Anna T. Cianciolo**, PhD, is Associate Professor of Medical Education at SIU School of Medicine.

**Debra L. Klamen**, MD, MHPE is Senior Associate Dean for Education and Curriculum and Chair of Medical Education at SIU School of Medicine.

**Austin M. Beason**, MD, and **Evyn L. Neumeister**, MD, are recent graduates of SIU School of Medicine’s undergraduate medical education curriculum.
