# Supplementary material for: ASPIRE-ing to Excellence at SIUSOM
Source: MedEdPublish (2016). 2017 May 5;6:82. [Version 1] doi: 10.15694/mep.2017.000082 (PMC10885253; doi:10.15694/mep.2017.000082)
Supplement: Peer review history

# Reviewer report: Reviewer response for version 1

**DOI:** 10.21956/mep.19296.r28101

**Published:** 2017-05-13

**Authors:** Peter Dieter

**Affiliations:** 1 AMSE

This review has been migrated. The reviewer awarded 5 stars out of 5 An exciting paper, well and clear written. It should stimulate ASPIRE winners to share their story as well as to stimulate other schools to join the ASPIRE family.

Reviewer Expertise:

NA

No decision status is available

# Reviewer report: Reviewer response for version 1

**DOI:** 10.21956/mep.19296.r28102

**Published:** 2017-05-07

**Authors:** Richard Hays

**Affiliations:** 1 James Cook University

This review has been migrated. The reviewer awarded 5 stars out of 5 I found this paper both interesting and enjoyable. It is a well written, clear description of both the process of applying for an ASPIRE award and a reflection on what made for success. Faculty at all schools considering applying should read this, because it makes it clear that success is wonderful, but not easy to achieve without a lot of effort, even at such a successful school. Perhaps there could have been more explicit coverage of 'what was learned' from both the success and the less successful episode. How useful was the feedback? As with most accreditation processes, the self-evaluation and preparation may be very helpful in developing a culture of continuing quality improvement.

Reviewer Expertise:

NA

No decision status is available

# Reviewer report: Reviewer response for version 1

**DOI:** 10.21956/mep.19296.r28100

**Published:** 2017-05-05

**Authors:** Trevor Gibbs

**Affiliations:** 1 AMEE

This review has been migrated. The reviewer awarded 5 stars out of 5 Recognition of excellence in medical and healthcare professional schools has always been a difficult subject to discuss and debate; the vast majority of schools showing"excellence" through research rather than their core objective- teaching and learning. The ASPIRE awards were designed to place the emphasis clearly upon teaching and learning with dumbing down to greatly the research element. This is a very well written reflective piece, written by a successful school. It describes an honest approach to the ASPIRE initiative and provides some helpful ideas should others wish to apply for the wards. I would recommend this paper to all those in leadership positions in healthcare and medical schools

Reviewer Expertise:

NA

No decision status is available

# Reviewer report: Reviewer response for version 1

**DOI:** 10.21956/mep.19296.r28103

**Published:** 2017-05-05

**Authors:** Ronald M Harden

**Affiliations:** 1 AMEE

This review has been migrated. The reviewer awarded 5 stars out of 5 I was impressed when I heard Anna talk at the AAMC Conference in Seattle in November on her school's experience with the ASPIRE to excellence awards . This paper is an extraordinarily powerful description of how a medical school can achieve and demonstrate excellence in medical education . I have given it a 5* rating as I believe that everyone engaged in educating doctors should read it.

Reviewer Expertise:

NA

No decision status is available
